# Influence of Source Materials, Concentration, Gastric Digestion, and Encapsulation on the Bioactive Response of Brassicaceae-Derived Samples against *Helicobacter pylori*

**DOI:** 10.3390/microorganisms12010077

**Published:** 2023-12-30

**Authors:** Paula Garcia-Ibañez, Jose Manuel Silvan, Diego A. Moreno, Micaela Carvajal, Adolfo J. Martinez-Rodriguez

**Affiliations:** 1 Grupo de Aquaporinas, Departamento de Nutrición Vegetal, Centro de Edafología y Biología Aplicada del Segura (CEBAS), Consejo Superior de Investigaciones Cientificas (CSIC), Campus Universitario de Espinardo, Edificio 25, 30100 Murcia, Spain; pgibanez@cebas.csic.es (P.G.-I.); mcarvaja@cebas.csic.es (M.C.); 2 Grupo de Microbiología y Biocatálisis de Alimentos (MICROBIO), Departamento de Biotecnología y Microbiología, Instituto de Investigación en Ciencias de la Alimentación (CIAL), CSIC-UAM (Consejo Superior de Investigaciones Cientificas-Universidad Autonoma de Madrid), C/Nicolas Cabrera 9, Universidad Autónoma de Madrid, 28049 Madrid, Spain; 3 Phytochemistry and Healthy Food Laboratory, Departamento de Ciencia y Tecnología de Alimentos, Centro de Edafología y Biología Aplicada del Segura (CEBAS), Consejo Superior de Investigaciones Cientificas (CSIC), Campus Universitario de Espinardo, Edificio 25, 30100 Murcia, Spain; dmoreno@cebas.csic.es

**Keywords:** isothiocyanates, *Helicobacter pylori*, Brassicaceae, antibacterial, antioxidant, anti-inflammatory

## Abstract

Isothiocyanates may have antibacterial activity against *Helicobacter pylori*, but there are different variables related to Brassicaceae-derived samples that could affect their efficacy. This work studied the influence of source variety, concentration, gastric digestion, and encapsulation of samples on their bioactive response against *Helicobacter pylori*. The antibacterial activity of raw sprouts (red cabbage and red radish) showed the highest antibacterial effect, which was consistent with a higher amount of isothiocyanates. It decreased with gastric digestion, regardless of sample encapsulation. By contrast, adult red radish leaves became antibacterial after gastric digestion. Antioxidant activity on *H. pylori*-infected gastric cells was similar in all samples and followed an equivalent pattern with the changes in isothiocyanates. Raw samples decreased intracellular reactive oxygen species production, but they lost this capacity after gastric digestion, regardless whether the compounds were free or encapsulated. Red cabbage sprouts, red radish sprouts, and red radish roots produced a decrease in nitric oxide production. It was consistent with a modulation of the inflammatory response and was associated to isothiocyanates concentration. Encapsulated sprout samples retained part of their anti-inflammatory activity after gastric digestion. Adult raw red radish leaves were not active, but after digestion, they became anti-inflammatory. The results obtained in this study have shown that several variables could have a significant impact on the bioactive properties of Brassicaceae-derived samples against *H. pylori*, providing a starting point for the design and standardization of samples with specific bioactivities (antibacterial, antioxidant, and anti-inflammatory) potentially useful for the treatment of *H. pylori* infection.

## 1. Introduction

*Helicobacter pylori* (*H. pylori*) is present in the stomach of approximately 50% of the global population [1,2]. This gram-negative bacterium has been reported to be able to establish a progressive chronic gastric inflammation, which can progress to clinical pathologies in 1 to 10% of individuals. In this way, *H. pylori* is the main bacterium responsible for several gastrointestinal diseases, such as peptic ulcer, gastric atrophy, and ultimately, gastric cancer [3,4]. *H. pylori* treatment has proven to be remarkably arduous, since no monotherapy has reported enough efficacy in its eradication and the antibiotics with higher effectiveness are limited (such as amoxicillin, clarithromycin, and tetracycline, among others). In clinical practice, antibiotics are often administered in combinations of two or three, along with an acid inhibitor or a bismuth component, to increase the antibiotic effect and provide mucosal protection [5,6]. Along with this limited treatment choice, *H. pylori* also has demonstrated an exceptional adaptation ability, being able to rapidly develop primary antibiotic resistance. This has led to a reduction in the success probabilities for the combined treatments and a great dilemma for patients that do not respond to consecutive drug therapies [7,8]. Therefore, since 2017, *H. pylori* has been included by the World Health Organization (WHO) among the 20 antibiotic-resistant pathogens that are supposed to be the most serious threat to human health [9]. In this way, recent interest is increasing not only in the research for new antibiotic therapies but also in the use of other compounds that could enhance antibacterial and anti-inflammatory activities, reducing therapy dosage and side effects. Throughout history, a relevant percentage of pharmacological drugs has been discovered in vegetal sources. In recent years, in vitro and in vivo studies have suggested that *H. pylori* infection can be inhibited through compounds found in fruits, vegetables, and spices [10,11]. In addition, some relevant medicinal plants and isolated compounds from herbs have been successfully evaluated for the treatment or eradication of *H. pylori* [12,13]. Recently, a high interest has been developed in the health benefits of food bioactives, with cruciferous vegetables standing out. Their benefit is based on a wide range of nutrients together with flavonols, anthocyanins, and glucosinolates (GSL). Cruciferous vegetable sprouts emerge as a great source of biomolecules because of their short production time and their high concentration of GSL, which are Brassicaceae-exclusive secondary metabolites that belong to the plant defense response [14]. Their main structure is a β-thioglucoside-N-hydrosulfate linked to a sulphur-β-glucopyranose, with a side chain that can vary depending on the precursor amino acid [15]. GSL are highly stable but they must undergo hydrolysation by the action of the myrosinase enzyme, in order to produce bioactive derivatives, such as isothiocyanates (ITC) and indoles [16]. Brassica sprouts (e.g., broccoli) present a high content of glucoraphanin, and thus, they can provide a high proportion of sulforaphane (SFN), to which diverse bioactivities have been attributed, such as anti-tumorigenic, antioxidant, anti-inflammatory, and antibacterial. According to these bioactive effects, diverse modes of action of ITC have been described, like influencing the membrane integrity, the inhibition of regulatory/enzymatic pathways, and the induction of resistance mechanisms to ITC [17]. Nevertheless, little to no information is found about the performance of this ITC from a complex matrix, such as the sprouts or adult plants, or in an enriched sample as a food-derived prototype. Recently, the use of encapsulating agents as nanocarriers has also reported good results for increasing ITC stability and delivery. Since these biomolecules can be affected by the low pH of the stomach, the use of Brassica-derived plasma membrane vesicles seems a feasible option to increase its stability in this adverse environment. Previous works performed with plasma membrane vesicles obtained from cauliflower inflorescences revealed high preservation of ITC in two different in vitro gastrointestinal digestion models [18]. Furthermore, works performed with broccoli-derived plasma membrane vesicles revealed an antiproliferative activity in melanoma cells per se, so they might also enhance other bioactivities of ITC [19]. In summary, the main purpose of this work was to determine the impact on the antibacterial, antioxidant, and anti-inflammatory properties against *H. pylori* of different variables (influence of source materials, concentration, gastric digestion, and encapsulation) that may affect the bioactive response of Brassicaceae-derived samples.

## 2. Materials and Methods

### 2.1. Plant Material

For obtaining the sprouts, 50 g of seeds from red cabbage (*Brassica oleracea* L. *var capitata f. rubra*) and red radish (*Raphanus sativus* L. var. sativus cv. Sango) were provided by Intersemillas S.A. (Loriguilla, Valencia, Spain). They were germinated after 24 h of decontamination in 1% bleach in deionized water and continuous aeration. Then, they were sown on cellulose as an inert substrate and stored in the dark for 48 h. After that, the three-day-old germinated seeds were placed in a growth chamber to grow the sprouts under a 20 W Protect BioLED (SysLed Spain, S.L., Madrid, Spain) and an established photoperiod of 18 h/6 h (light/darkness), a temperature cycle of 24/18 °C, and a relative humidity of 60–80%. After reaching 8 days of growth, the sprouts were freeze-dried and grounded, followed by storage at −80 °C. For obtaining the adult red radishes (*Raphanus sativus* L.), thirty commercial seeds were supplied by SAKATA Seed Ibérica S.L.U. (Valencia, Spain), and were induced to germinate by hydration under continuous aeration using deionized water for 24 h. After the initial imbibition, the seeds were transferred to vermiculite substrate trays and left germinating in the dark for 2 days at 37 °C with high relative humidity (80%). The 3-day-old sprouts were transplanted to hydroponic containers with complete Hoagland’s Nutrient Solution prepared as available elsewhere [20]. At a stage of five leaves growth (21-day-old plants), the plants were harvested, and the leaves were processed separately from the roots (including the edible bulbs). Samples were freeze-dried and powdered and kept at −80 °C until further analysis.

### 2.2. Cauliflower-Derived Plasma Membrane Purification

One hundred g of cauliflower inflorescences (*Brassica oleracea* L. var. botrytis), kindly provided by SAKATA Seed Ibérica S.L.U., was processed as described in Garcia-Ibañez et al. [18]. For purity determination, the final protein concentration was measured using an RC DC protein assay kit (BioRad, Hercules, CA, USA) and using bovine serum albumin as a standard (Sigma-Aldrich, St. Louis, MA, USA).

### 2.3. Sample Preparation and Gastric In Vitro Digestion Process

Aqueous samples (1:15, *w*/*v*) from both red cabbage sprouts, red radish sprouts, and leaves/roots of the adult plant of red radish were obtained by maceration during 4 h in darkness and under continuous agitation. After centrifugation at 13,500× *g* for 15 min, the supernatants were collected and percolated through a qualitative filter. For the nanoencapsulated treatments, a final protein concentration of 0.2% of the vesicles was added to 5 mL of each extract. Then, samples were vortexed for 2 min for membrane disruption. Afterwards, samples were introduced in ice for membrane reconstitution. For the in vitro gastric digestion, 1 mL of each sample (free and nanoencapsulated) was digested with 15 mL of a porcine pepsin solution of 200 U/mL in 100 mM HCl at pH 2 (Sigma-Aldrich). Samples were kept in this fluid for 3 h at 37 °C. Subsequently, pH was neutralized to 7 with 7 mL of 0.2 M sodium hydroxide (NaOH). Samples were kept at 4 °C and filtered through a 0.22 µm pore diameter of PVDF and then freeze-dried for further assays.

### 2.4. Isothiocyanates Identification in the Gastric Digested Treatments

The quantitative and qualitative determination of ITC was performed using a UHPLC coupled to a 6460-triple quadrupole-MS/MS (Agilent Technologies, Waldbronn, Germany) and with a reverse phase column C18 Zorbax Eclipse Plus (2.1 × 50 mm, 1.8 µm) [21]. For quantification, sulforaphane, indole-3-carbinol (I3C), and 3,3-diindolylmethane from Santa Cruz Biotech (Santa Cruz, CA, USA) were used as standards. In order to determine each compound of interest, retention times and fragmentation patterns were studied.

### 2.5. Growth and Culture Conditions of H. pylori

*H. pylori* strain (Hp59) was obtained from the MICROBIO group bacterial collection (CIAL). *H. pylori* strain was stored in Brucella Broth (BB) medium (Becton, Dickinson, & Co., Madrid, Spain) supplemented with 20% (*v*/*v*) glycerol and placed at −80 °C until use. The agar-plating medium used was Müeller-Hinton agar supplemented with 5% defibrinated sheep blood (MHB) (Becton, Dickinson, & Co.). A liquid medium BB supplemented with 10% horse serum (HS) (Biowest, Barcelona, Spain) was used. The inoculum for *H. pylori* strain was prepared as described by Silvan et al. [22] and it can be summarized as follows: frozen strain was reactivated by inoculation (200 μL) in MHB plate and incubation in a Variable Atmosphere Incubator (VAIN) (85% N_2_, 10% CO_2_, and 5% O_2_) (MACS-VA500, Don Whitley Scientific, Bingley, UK) at 37 °C for 72 h. Bacteria grown from one MHB plate was suspended in 2 mL of BB + 10% HS or culture medium cell (~1 × 10^8^ colony forming units (CFU/mL)) and used as bacterial inoculum in the different assays.

### 2.6. Cell Culture Conditions

Human gastric epithelial (AGS) and murine macrophage (RAW264.7) cell lines were obtained from the American Type Culture Collection (ATCC, Manassas, VA, USA). Cells were cultured in Dulbecco’s Modified Eagle’s Medium-F12 (DMEM/F12) (Lonza, Madrid, Spain) supplemented with 10% (*v*/*v*) fetal bovine serum (FBS) (Hyclone, GE Healthcare, Logan, UK) and 1% (*v*/*v*) penicillin/streptomycin (5000 U/mL) (Lonza). Cell cultures and subcultures were prepared as described by Silvan et al. [23]. Briefly, cells were plated at densities of ~1 × 10^6^ cells in 75 cm^2^ culture flasks (Sarstedt, Barcelona, Spain) and maintained at 37 °C under 5% CO_2_ in a humidified incubator until 90% of cell confluence. The cell culture medium was changed every 2 days. Before a confluent monolayer appeared, cell sub-culturing was carried out. All experiments were carried out between passage 10 and passage 30 to ensure cell uniformity and reproducibility.

### 2.7. Cell Viability

The cytotoxicity of the digestates from red cabbage and red radish sprouts and the leaves and roots of adult red radish plants (free and nanoencapsulated), as well as the raw samples, was evaluated. Cell lines viability (AGS and RAW264.7) was determined by MTT (3,4,5-dimethylthiazol-2,5-diphenyl-tetrazolium bromide) (Sigma Aldrich) reduction assay following a protocol previously described [23]. Briefly, confluent cell cultures (~90%) were trypsinized (Trypsin/EDTA 170,000 U/L) (Lonza) and cells were seeded (~5 × 10^4^ cells per well) in 96-well plates (Sarstedt) and incubated in complete cell culture medium at 37 °C under 5% CO_2_ in a humidifier incubator for 24 h. Complete cell culture medium was replaced with serum-free culture medium containing the non-digested and digested samples (at 1.5 and 2 mg/mL final concentration), and cells were incubated at 37 °C under 5% CO_2_ for 2 h. The control cellular group was incubated in serum-free medium without samples. Thereafter, cells were washed with phosphate-buffered saline (PBS) (Lonza), the medium was replaced by 180 μL of serum-free medium, and 20 μL of MTT solution in PBS (5 mg/mL) was added to each well for the quantification of the living metabolically active cells after incubation at 37 °C under 5% CO_2_ in a humidifier incubator for 1 h. Yellowish MTT is reduced to purple formazan crystals in the mitochondria of metabolically active cells. Formazan crystals in the wells were solubilized in 200 μL dimethyl sulfoxide (Sigma). Finally, absorbance was measured at 570 nm wavelength using a microplate reader Synergy HT (BioTek Instruments Inc., Winooski, VT, USA). The viability was calculated considering the control cellular group (non-treated) as 100% of viability. Results were obtained from three independent experiments (n = 3).

### 2.8. Determination of the Antibacterial Activity of the Brassicaceae-Derived Samples against H. pylori

The antibacterial activity of non-digested (raw) and digested samples from the different types of Brassicaceae (red cabbage/red radish sprouts and leaves/roots of red radish adult plants) against *H. pylori* was determined following the protocol established by Silvan et al. [22]. Briefly, 1 mL of each sample (at 2 mg/mL final concentration) dissolved in sterile water was transferred into a flask with 4 mL of BB supplemented with 10% HS. The bacterial inoculum (100 µL of ~1 × 10^8^ CFU/mL) was inoculated into the flasks under aseptic conditions. Cultures were incubated with continuous stirring (150 rpm) in a microaerophilic atmosphere in a VAIN at 37 °C for 24 h. Positive growth control was performed by adding 1 mL of sterile water to 4 mL of BB supplemented with 10% HS and 100 µL of bacterial inoculum. BB supplemented with 10% HS was used as negative growth control. Afterwards, serial decimal dilutions of *H. pylori* cultures were prepared in saline solution (0.9% NaCl) and, subsequently, 200 µL was plated onto fresh MHB agar for further incubation in a microaerophilic atmosphere using a VAIN at 37 °C for 72 h. The number of CFU was assessed after incubation. Results of antibacterial activity were expressed as Log CFU/mL (n = 3). The Minimal Inhibitory Concentration (MIC) of non-digested (raw) and digested samples was determined following the procedure described above. Samples were diluted in BB to obtain the desired final concentrations. MIC was defined as the lowest amount of sample that provoked a significant (*p* < 0.05) decrease in viability compared with the control growth after 24 h of treatment [23]. The dilution intervals for determination of MIC ranged from 2 to 0.1 mg/mL.

### 2.9. Determination of the Antioxidant Activity of the Brassicaceae-Derived Samples against Intracellular Reactive Oxygen Species (ROS) Production by AGS Cells

The human gastric epithelial AGS cell line was used for the evaluation of oxidative stress induced by tert-butyl hydroperoxide (T-BHP). The measurement of intracellular ROS was performed by DCFH-DA (carboxy-2′,7′-dichloro-dihydrofluerescein diacetate) (Sigma) assay as reported in Silvan et al. [24]. Cells were seeded (~5 × 10^4^ cells per well) in 24-well plates and incubated at 37 °C under 5% CO_2_ for 24 h. For treatments, cells were incubated with the raw samples and the gastric digestates (1.5 mg/mL) reconstituted in serum-free medium at 37 °C under 5% CO_2_ for 120 min. After that, cells were washed with PBS and incubated with 20 μM DCFH-DA dissolved in serum-free culture medium at 37 °C under 5% CO_2_ for 30 min. Then, cells were washed twice with PBS to remove the unabsorbed probe and treated with serum-free culture medium containing 2.5 mM T-BHP (pro-oxidant agent). ROS production was immediately monitored for 180 min in a fluorescent microplate reader Synergy HT (BioTek Instruments Inc.) using a λ_ex_485 nm and λ_em_530 nm. After being oxidized by intracellular oxidants, DCFH-DA changes to dichlorofluorescein (DCF) and emits fluorescence. Non-treated cells were used as basal control (C−) (100% of intracellular ROS production). Cells incubated with T-BHP were used as positive oxidation control (C+). All samples were analyzed in triplicate (n = 3). Results were expressed as % of inhibition of ROS production.

### 2.10. Determination of the Anti-Inflammatory Activity of the Brassicaceae-Derived Samples

For the anti-inflammatory activity evaluation, murine macrophage cell line RAW264.7 was used. The assay was carried out following the procedure described by Silvan et al. [24]. Briefly, cells were seeded (~5 × 10^4^ cells/well) in 96-well plates (Sarstedt) and incubated in complete cell culture medium at 37 °C under 5% CO_2_ in a humidifier incubator for 24 h. For treatments, cells were incubated with the raw samples and the gastric digestates (1.5 mg/mL) were reconstituted in serum-free medium at 37 °C under 5% CO_2_ for 120 min. Cells were washed with PBS and treated with 10 μg/mL of LPS from *Escherichia coli* O55:B5 dissolved in serum-free culture medium and incubated at 37 °C under 5% CO_2_ for 24 h. Control cellular groups were incubated in serum-free culture medium with LPS (inflammation control group) (C+) or without LPS (no-inflammation control group) (C−) for 24 h. Finally, cellular supernatants were collected, particulate material was removed by centrifugation (12,000 rpm for 10 min), and samples were stored at −20 °C until analysis was performed. Nitrite accumulation, an indicator of nitric oxide (NO) synthesis as an inflammatory biomarker, was measured in the cellular supernatants by the Griess reaction. Briefly, 100 μL of cellular supernatants was plated in a 96-well plate and an equal amount of Griess reagent constituted by 1% (*w*/*v*) sulfanilamide and 0.1% (*w*/*v*) N-(1-naphthyl)ethylenediamine-dihydrochloride in 2.5% (*v*/*v*) H_3_PO_4_ was added. The plate was incubated for 5 min and the absorbance was measured at 550 nm in a microplate reader Synergy HT (Biotek Instruments Inc.). The amount of NO was calculated using a sodium nitrite standard curve ranging from 0 to 15 μg/mL. Data were expressed as percentage of NO production calculated relative to the inflammation control group (100% of inflammation). Data represent the mean and standard deviation of three independent experiments (n = 3).

### 2.11. Statistical Analysis

The results were reported as mean values ± standard deviations (SD) performed at least in triplicate (n = 3). Significant differences were estimated by applying *t*-test or variance analysis (ANOVA) as required. The Tukey’s least significant differences (HSD) test was used to evaluate the significance of these values. In all cases, differences were considered significant at *p* < 0.05. All statistical tests were performed with IBM SPSS software Statistics for Windows, Version 26.0 (IBM Corp., Armonk, NY, USA).

## 3. Results

### 3.1. Raw Samples and Digestates Characterization

The qualitative and quantitative characterization of ITC of the sample is shown in Table 1. In red cabbage sprouts, erucin was only quantified in the raw sample, but it was also present in traces in the two gastric digestates (free and nanoencapsulated). Iberin was the major compound in red cabbage sprout samples, with the highest concentrations in the raw sample (190 µM). Nanoencapsulation protected around 16% iberin degradation with respect to the non-encapsulated sample. The concentration of indole-3-carbinol (I3C) sharply decreased to negligible levels after the digestion process, and nanoencapsulation was only able to retain 3.8% of this compound.

High levels of SFN (141 µM) were quantified in the raw sample, but a significant decrease was observed after the digestion process, regardless of the encapsulation. Sulforaphene was absent in these samples. In red radish sprouts, erucin was quantifiable in the three sample types, but the highest concentration was found in the nanoencapsulated gastric digestate (0.47 µM; *p* < 0.05). Iberin was significantly affected (*p* < 0.05) by gastric digestion, which caused a drastic decrease in its concentration in both samples (nanoencapsulated and non-encapsulated), although degradation was greater in the non-encapsulated sample, where iberin was only found in trace levels and it was not possible to quantify it. Surprisingly, I3C was only quantified in the raw sample because gastric digestion reduced its presence to mere traces in the digested samples. SFN and sulforaphane also decreased significantly (*p* < 0.05) during gastric digestion. However, nanoencapsulation increased sulforaphene biodisponibility when compared to the free gastric digestion (*p* < 0.05). In the adult red radish leaves, erucin and iberin were not detected in any of the samples, while I3C followed a similar pattern to the previous compounds. Gastric digestion provoked a decrease of more than 95% of I3C with significantly lower degradation of nanoencapsulated samples (0.45 µM) compared to non-encapsulated ones (0.08 µM). Similar results were obtained for SFN and sulforaphene, which showed higher concentrations in the raw samples (*p* < 0.05) when compared to the digestates. In the case of sulforaphene, the results were similar for the two digests, regardless of encapsulation. Finally, in the reed radish root, erucine was the major compound identified and no significant differences (*p* < 0.05) were observed between the raw sample and the digestate nanoencapsulated, while the non-encapsulated sample had a degradation of 58.5% compared to the nanoencapsulated one. Iberin was not identified and I3C was not quantified after gastric digestion, but 57.4% of I3C was protected from degradation after nanoencapsulation. Similar behavior was observed for SFN and sulforaphene. Nanoencapsulation protected 83.3% and 80% from degradation, respectively.

### 3.2. Antibacterial Activity of Brassicaceae-Derived Samples and Digestates against H. pylori

The antibacterial activity of the samples against *H. pylori* is shown in Table 2. It can be seen that the antibacterial activity was affected by all the variables analyzed (source of ITC compounds, digestive process, encapsulation, and sample concentration). At the higher concentration (2 mg/mL), all red cabbage sprout samples produced a significant reduction in bacterial growth (*p* < 0.05). Raw and encapsulated gastric digested samples fully suppressed *H. pylori* growth, showing a bactericidal effect (<1.5 log CFU/mL), while the non-encapsulated gastric digest was less active, reducing *H. pylori* growth 4.74 log CFU/mL, showing that encapsulation protected some ITC compounds with antibacterial activity from degradation, as shown in Table 1. However, the digestion process, independent of encapsulation, decreases ITC concentration (Table 1), and its impact on antibacterial activity was observed when the antibacterial activity was measured at lower concentrations. In these cases, the MIC value of raw samples (0.2 mg/mL) was ten times lower than in gastric digested samples (2 mg/mL). The three samples of red radish sprouts showed a bactericide effect against *H. pylori* at the higher concentration (2 mg/mL). However, as described above, the raw sample has the highest bactericidal effect at lower concentrations, showing an MIC value of 0.1 mg/mL, 20-fold lower than for the digested sample (2 mg/mL), also evidencing the detrimental effect of digestion on antibacterial activity against *H. pylori*. Samples from red radish leaves showed a completely different behavior. In this case, the raw sample did not have antibacterial activity against *H. pylori*, while the gastric digests, regardless of encapsulation, were bactericidal and with identical MIC, indicating that compounds with antibacterial activity against *H. pylori* are produced during the digestion process. This is not particularly evident from the analysis of ITC composition (Table 1), suggesting that compounds other than ITC are involved in the antibacterial effect against *H. pylori* and they will require further studies. Finally, red radish root showed no significant (*p* > 0.05) antibacterial activity against *H. pylori*, either as a raw sample or after gastric digestion, in agreement with the fact that in this case, ITC compounds were in very low concentrations or absent (erucin) in all samples.

### 3.3. Antioxidant Activity of Brassicaceae-Derived Samples and Digestates against Intracellular Reactive Oxygen Species (ROS) Production by AGS Cells

**Figure 1:**
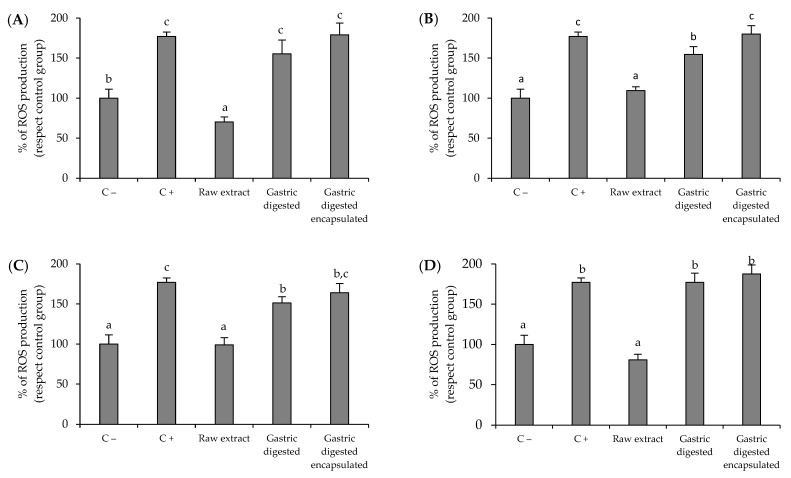
Effects of red cabbage sprout (**A**), red radish sprout (**B**), red radish leaves (**C**), red radish root (**D**), and their digests on reactive oxygen species (ROS) production by AGS cells. Basal ROS production by AGS cells (C−, negative control). ROS production by AGS treated with T-BHP (pro-oxidant agent) (C+, positive control). Histograms represent the relative production of ROS with respect to non-oxidized control cells (C−, negative control) (100% of ROS production) after 2 h of pre-incubation with the samples (1.5 mg/mL) and subsequent treatment with T-BHP (pro-oxidant agent) (C+, positive control) for 3 h. Data show the mean ± SD (n = 3). ^a–c^ Bars with different letters indicate significant differences according to ANOVA (*p* < 0.05). Post hoc test: Tukey Test.

The results of the ROS scavenging assay performed on gastric AGS cells are shown in Figure 1. The experiments were conducted at a sample concentration of 1.5 mg/mL, the highest sample concentration which did not significantly affect the viability of AGS gastric cells. Cells exposed to T-BHP pro-oxidant agent (C+) significantly increased the ROS production (*p* < 0.05) with respect to non-oxidized control cells (C−). After treatment with the raw red cabbage sprout, there was a significant reduction in ROS production (*p* < 0.05) to values lower than those obtained for non-stimulated AGS cells (70.3% and 100%, respectively), demonstrating the high antioxidant potential of the raw red cabbage sprout sample. However, this effect disappeared with the digested samples (Figure 1A), regardless of encapsulation. Similar results were obtained for the rest of the sources of ITC: red radish sprout (Figure 1B), red radish leaves (Figure 1C), and red radish root (Figure 1D), in which the raw samples reduced ROS production to a similar level to that the non-stimulated control, while gastric digestion significantly affected this behavior. This response is related to the decrease in ITC concentration during digestion (Table 1), suggesting that the antioxidant activity of ITCs on AGS gastric cells is dramatically affected by the digestion process regardless of the sample encapsulation, although when raw samples are considered, the antioxidant activity is independent of the source of ITC used.

### 3.4. Anti-Inflammatory Activity of Brassicaceae-Derived Samples and Digestates

**Figure 2:**
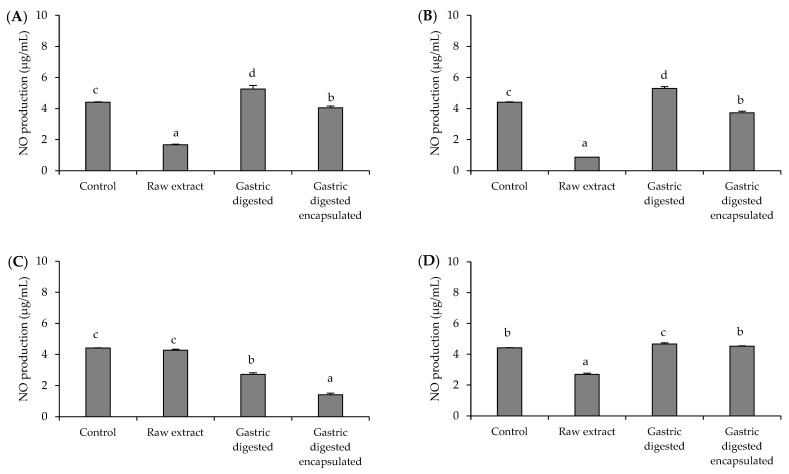
Effect of red cabbage sprout (**A**), red radish sprout (**B**), red radish leaves (**C**), red radish root (**D**), and their digests (1.5 mg/mL) on nitric oxide (NO) production in LPS-stimulated RAW264.7 macrophage cells. NO production by RAW264.7 treated with LPS (pro-inflammatory agent) (C+, positive control). Histograms represent the relative production of NO with respect to LPS-stimulated cells (LPS) (100% production) (C+, positive control) after 2 h of incubation with the samples (1.5 mg/mL) and subsequent stimulation with LPS for 24 h. Data are represented by mean ± SD (n = 3). ^a–d^ Bars with different letters indicate significant differences in NO production according to ANOVA *(p* < 0.05). Post hoc test: Tukey Test.

Results of the NO production in the LPS-stimulated RAW264.7 macrophage cells are shown in Figure 2. Similar to AGS cells, 1.5 mg/mL was used as the experimental concentration for samples, since it was the highest concentration that did not affect the viability of RAW264 cells. When cells were treated with sprout samples (red cabbage sprout (Figure 2A) and red radish sprout (Figure 2B)), the behavior was quite similar. The red cabbage and red radish raw samples significantly reduced NO production (*p* < 0.05) to levels lower than those obtained for the control (37.7% and 19.6%, respectively), while gastric digestion suppressed this effect, although encapsulation protected in some degree the capacity in these samples to reduce NO production in the cells. This behavior is consistent with the changes in ITC compounds during digestion (Table 1). The inverse effect was observed for the red radish leaves sample (Figure 2C). In this case, the raw sample did not affect LPS-stimulated cells, whereas gastric digests decreased cellular NO production. This decrease was higher in the case of the encapsulated sample than in the gastric digested sample (68% and 38.5% of NO reduction, respectively), showing that the anti-inflammatory capacity is enhanced by gastric digestion. Similar to the results of the antibacterial activity, the behavior of the red radish leaves sample was different and did not coincide with the variations observed for the ITC compounds (Table 1), indicating that other compounds seem to be involved and need further research. Finally, the raw sample of red radish root caused a 39.1% reduction in cellular NO production (Figure 2D), while after gastric digestion, this effect vanished, resulting in NO production values similar to those obtained for cells stimulated with LPS. In this last case, the two samples had similar responses during digestion, regardless of the sample encapsulation.

## 4. Discussion

The emergence of antibiotic-resistant strains has complicated the management of *H. pylori* infection, highlighting the need for the development of new treatment strategies. These new strategies are intended not only at the search for compounds with antibacterial activity against *H. pylori*, but also with the potential to modulate the oxidative and inflammatory damage that results in gastric epithelial cells when they are infected with *H. pylori*. Modulation of the oxidative and inflammatory response in the gastric epithelium has been shown to be particularly relevant in preventing tissue damage and the progression of pathologies associated with *H. pylori* infection [25,26]. In the present work, raw samples and their free and nanoencapsulated gastric digestates from different Brassicaceae-derived samples were assessed to evaluate their potential bioactivities (antibacterial, antioxidant, and anti-inflammatory) against *H. pylori* and the impact of the different variables evaluated (source of ITC compounds, digestive process, encapsulation, and sample concentration) on the obtained response. Generally, the highest concentrations of ITC were found in raw samples of the four biological sources (Table 1). Both digested samples (free and nanoencapsulated) were impaired by gastric digestion when compared to the raw sample. However, this effect was not the same in both cases. As described by others [27], the nanoencapsulation in cauliflower plasma membrane vesicles contributed to preserving a fraction of the ITC compounds. The difference in ITC composition between raw and digested (free or nanoencapsulated) samples influenced their bioactive response against *H. pylori*. This response was related both to the type of bioactivity assayed (antibacterial, antioxidant, and anti-inflammatory) and to the different source materials used (red cabbage sprouts, red radish sprouts, adult red radish leaves, and adult red radish roots). The antibacterial activity against *H. pylori* was different when sprouts or adult plants were used as a source of ITC. For antibacterial activity, raw samples from sprouts (red cabbage and red radish) showed the highest antibacterial effect (MIC of 0.2 and 0.1 mg/mL, respectively), which is consistent with the presence of the higher ITC concentration in sprout samples. Although several ITC compounds could be involved in the antibacterial activity, SFN, which was especially abundant in the raw samples from Brassicaceae sprouts (Table 1), has been shown to be particularly effective as an antibacterial against *H. pylori* both in vitro and in different clinical trials [28,29]. It has also been demonstrated that this compound is capable of inhibiting up to 36% of urease activity, an enzyme that constitutes one of the main virulence attributes of *H. pylori* [30]. The effect of gastric digestion on the antibacterial activity of the sprouts depended on both the type of sprout (red cabbage or red radish) and the concentration of the sample. Encapsulation only preserved the antibacterial response of red cabbage sprouts when it was used at the highest concentration (2 mg/mL). Adult plants had a completely different behavior. While the raw sample from radish leaves did not show antibacterial activity, the digests did, regardless of whether they were nanoencapsulated or not, suggesting the participation of non-ITC compounds in the antibacterial response. For instance, the fiber in the sample after digestion produces the release of potential antibacterial compounds that could be retained in them, such as other phenolics not determined in this work, which are present in adult radish leaves and that have previously demonstrated their efficacy as antibacterial compounds against *H. pylori* [31]. Finally, none of the red radish root samples had antibacterial activity against *H. pylori*. These samples had the lowest concentration of ITC (except erucin) (Table 1), which could explain in part the obtained response. The lack of antibacterial activity reported in some samples could be due to the way the sample was prepared for analysis. The use of extraction green solvents other than pure water could facilitate the extraction of compounds responsible for antibacterial activity and should be explored in future work. In contrast to the antibacterial activity, the antioxidant capacity of the samples on *H. pylori*-infected gastric cells was higher in all raw samples, regardless of the source materials (red cabbage sprouts, red radish sprouts, adult red radish leaves, and adult red radish roots) (Figure 1). Samples lost this capacity after gastric digestion, regardless whether the compounds were in free or nanoencapsulated form. This behavior seems to be related to the decrease in ITC concentration produced during gastric digestion (Table 1). Compounds such as SFN can reduce ROS formation in a concentration-dependent way and protect gastric cells against induced oxidative stress [32,33]. The persistent colonization by *H. pylori* provokes a chronic mucosal inflammatory process, in which activated neutrophils and macrophages are implied. Their activation also entails an increase in ROS production at the inflammation site [34,35]. According to diverse studies, an increase in NO and its related gene (nitric oxide synthase) has been reported after *H. pylori* infection [36]. In this way, this parameter has been consistently related to patients with *H. pylori* and several mucosal inflammatory changes have been linked with a high NO luminal concentration [37]. The obtained results (Figure 2) showed that three raw samples (red cabbage sprouts, red radish sprouts, and red radish roots) produced a decrease in NO production, which is consistent with a modulation of the inflammatory response in gastric cells [38]. When these samples were digested, the anti-inflammatory effect decreased. Except for the red radish roots sample (sample with the lowest quantity of ITCs), a slight protective effect was observed in the nanoencapsulated samples. As also happened for the antibacterial activity, adult red radish leaves (Figure 2C) had a singular behavior, different from the rest of the samples. The raw sample failed to inhibit NO production, while the digested sample did. As described above, this behavior does not appear to be related to changes in ITC concentration and requires further study.

## 5. Conclusions

The present work has demonstrated that the bioactive properties of Brassicaceae-derived samples against *H. pylori* depend on several factors including the type of source material, the bioactive response to be promoted, the sample concentration, the effect of gastric digestion, and the sample encapsulation. Antibacterial activity was higher in the raw sprouts and was directly associated with the ITC concentration. Samples of raw adult plants had no antibacterial activity.

Gastric digestion decreased the antibacterial activity of the raw sprouts in most cases, regardless of sample encapsulation. However, samples of adult red radish leaves, inactive in their raw form, became antibacterial after gastric digestion. Antioxidant activity was similar in all samples and followed an equivalent pattern with the changes in ITCs concentration after digestion. Encapsulation did not influence the antioxidant behavior of the samples during gastric digestion. Anti-inflammatory activity showed a similar response to the antibacterial activity. It was higher in the raw sprouts than in adult plants and it was associated to ITC concentration. Except for the red radish root sample, the anti-inflammatory activity disappeared after gastric digestion. This work should provide a starting point for the design of Brassicaceae-derived samples with specific bioactivities (antibacterial, antioxidant, and anti-inflammatory) potentially useful in the treatment of *H. pylori* infection as an alternative treatment option or as an adjuvant to antibiotic treatment. All this knowledge will contribute in the future to standardize the production of specific formulations against *H. pylori*, thus avoiding different responses from apparently comparable samples, and promoting a similar behavior regardless of the variations inherent to the materials used for their production.

## Figures and Tables

**Table 1:** Isothiocyanates (ITC) quantification by UHPLC-ESI-QqQ-MS/MS of raw and digested samples (µM) form red cabbage sprouts, red radish sprouts, and red radish adult plant leaves and roots.

Samples	Erucin	Iberin	Indole-3-carbinol (I3C)	Sulforaphane (SFN)	Sulforaphene
*Red cabbage sprout*					
Raw	0.22 ± 0.01	190.00 ± 17.00 ^a^	4.38 ± 0.67 ^a^	141.22 ± 12.90 ^a^	-
Gastric digested	*	75.17 ± 0.57 ^c^	*	11.83 ± 0.11 ^b^	-
Gastric digested encapsulated	*	88.95 ± 2.34 ^b^	0.17 ± 0.05 ^b^	14.05 ± 0.10 ^b^	-
*Red radish sprout*					
Raw	0.11 ± 0.02 ^b^	6.74 ± 1.50 ^a^	4.04 ± 0.70	10.60 ± 1.30 ^a^	9.51 ± 1.01 ^a^
Gastric digested	0.14 ± 0.04 ^b^	*	*	0.95 ± 0.08 ^b^	0.37 ± 0.01 ^c^
Gastric digested encapsulated	0.47 ± 0.02 ^a^	0.73 ± 0.07 ^b^	*	0.80 ± 0.10 ^b^	0.81 ± 0.07 ^b^
*Red radish leaves*					
Raw	-	-	11.10 ± 1.02 ^a^	0.56 ± 0.03 ^a^	2.51 ± 0.07 ^a^
Gastric digested	-	-	0.08 ± 0.02 ^c^	0.06 ± 0.01 ^c^	0.10 ± 0.01 ^b^
Gastric digested encapsulated	-	-	0.45 ± 0.03 ^b^	0.10 ± 0.01 ^b^	0.13 ± 0.04 ^b^
*Red radish root*					
Raw	1.02 ± 0.20 ^a^	-	0.47 ± 0.10 ^a^	0.06 ± 0.00 ^a^	0.10 ± 0.03 ^a^
Gastric digested	0.55 ± 0.05 ^b^	-	*	0.01 ± 0.00 ^c^	0.04 ± 0.00 ^c^
Gastric digested encapsulated	0.94 ± 0.04 ^a^	-	0.27 ± 0.03 ^b^	0.05 ± 0.01 ^b^	0.08 ± 0.00 ^b^

(*) The isothiocyanate was present under the limit of quantification (LOQ < 0.0625 µM). (-) The isothiocyanate was absent. Data represent the mean ± SD (n = 3). Different letters indicate statistically significant differences in the ANOVA and HSD Tukey as a post hoc test between the different sample types of a biological material (*p* < 0.05).

**Table 2:** Antibacterial activity of raw and digested samples (at 2 mg/mL) on the viable counts of *H. pylori* strain after 24 h of treatment. Results are expressed as log Colony Forming Units (CFU)/mL ± SD (n = 3).

Samples	Log CFU/mL	Log Reduction	MIC (mg/mL)
*Red cabbage sprout*			
Raw	<1.5 *	>7.05	0.2
Gastric digested	3.82 ± 0.11 *	4.74	2.0
Gastric digested encapsulated	<1.5 *	>7.05	2.0
*Red radish sprout*			
Raw	<1.5 *	>7.05	0.1
Gastric digested	<1.5 *	>7.05	2.0
Gastric digested encapsulated	<1.5 *	>7.05	2.0
*Red radish leaves*			
Raw	8.47 ± 0.04	0.08	-
Gastric digested	<1.5 *	>7.05	1.0
Gastric digested encapsulated	<1.5 *	>7.05	1.0
*Red radish root*			
Raw	7.26 ± 0.05	1.29	-
Gastric digested	8.59 ± 0.02	-	-
Gastric digested encapsulated	8.62 ± 0.03	-	-

Control growth 8.55 log CFU/mL (positive control). CFU detection limit (bactericidal effect) was 1.5 log CFU/mL (30 CFU per plate). Values marked with asterisk indicate significant differences compared to the control growth by *t*-test (*p* < 0.05).

## Data Availability

Data are contained within the article.
